# Mitophagy-related regulated cell death: molecular mechanisms and disease implications

**DOI:** 10.1038/s41419-024-06804-5

**Published:** 2024-07-16

**Authors:** Molin Yang, Xiang Wei, Xin Yi, Ding-Sheng Jiang

**Affiliations:** 1 Division of Cardiovascular Surgery, Tongji Hospital, Tongji Medical College, Huazhong University of Science and Technology, Wuhan, Hubei China; 2 Key Laboratory of Organ Transplantation, Ministry of Education; NHC Key Laboratory of Organ Transplantation; Key Laboratory of Organ Transplantation, Chinese Academy of Medical Sciences, Wuhan, Hubei China; 3 Department of Cardiology, Renmin Hospital of Wuhan University, Wuhan, Hubei China

**Keywords:** Mitophagy, Cell death

## Abstract

During oxidative phosphorylation, mitochondria continuously produce reactive oxygen species (ROS), and untimely ROS clearance can subject mitochondria to oxidative stress, ultimately resulting in mitochondrial damage. Mitophagy is essential for maintaining cellular mitochondrial quality control and homeostasis, with activation involving both ubiquitin-dependent and ubiquitin-independent pathways. Over the past decade, numerous studies have indicated that different forms of regulated cell death (RCD) are connected with mitophagy. These diverse forms of RCD have been shown to be regulated by mitophagy and are implicated in the pathogenesis of a variety of diseases, such as tumors, degenerative diseases, and ischemia‒reperfusion injury (IRI). Importantly, targeting mitophagy to regulate RCD has shown excellent therapeutic potential in preclinical trials, and is expected to be an effective strategy for the treatment of related diseases. Here, we present a summary of the role of mitophagy in different forms of RCD, with a focus on potential molecular mechanisms by which mitophagy regulates RCD. We also discuss the implications of mitophagy-related RCD in the context of various diseases.

## Introduction

**Fig. 1:**
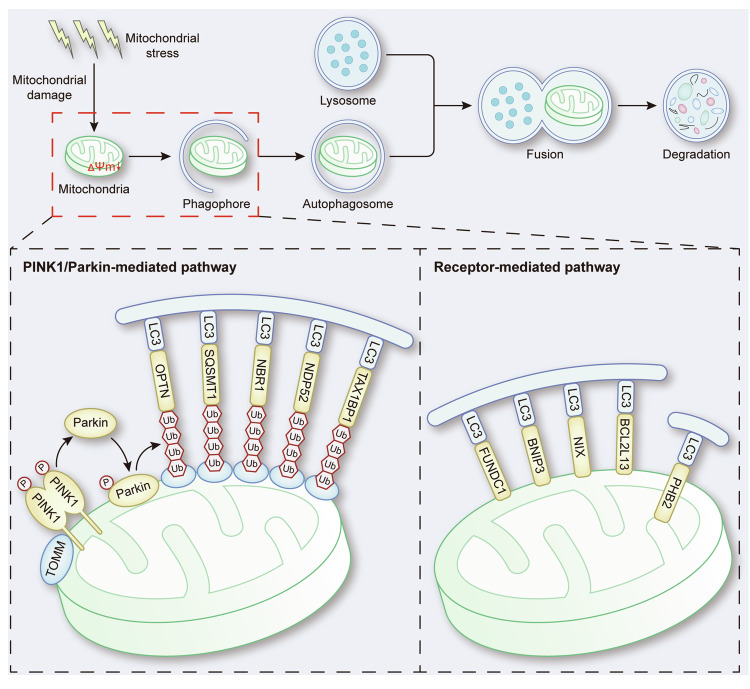
The molecular mechanisms of mitophagy. In response to mitochondrial damage, mitochondria undergo depolarization, leading to the stabilization of PINK1 on the OMM and the subsequent recruitment and phosphorylation of Parkin. Activated Parkin promotes the polyubiquitination of OMM proteins and recruits mitophagy receptors including OPTN, SQSMT1, NBP1, NDP52 and TAX1BP1, which bind with LC3 on the phagophore to activate mitophagy. Additionally, other mitophagy receptors such as FUNDC1, BNIP3, BNIP3L on the OMM, and PHB2 on the IMM directly interact with LC3 on the phagophore, thereby facilitating mitophagy.

The selective clearance of mitochondria through autophagy, which is known as mitophagy, is an important mechanism of mitochondrial quality control [1]. Through ubiquitin-dependent or ubiquitin-independent receptor-mediated pathways, damaged mitochondria are enclosed in autophagosomes and transported to lysosomes for degradation and recycling, which is important for basal mitochondrial turnover and the maintenance of physiological conditions [2, 3] (Fig. 1).

Currently, there is controversy regarding the role of mitophagy in diseases. Some studies have indicated that mitophagy can prevent disease progression by exerting a cytoprotective role. In osteoarthritis (OA), mitophagy can regulate reactive oxygen species (ROS) levels and increase the survival of human chondrocytes [4]. Additionally, impaired mitophagy has been reported to be related to neuronal loss in neurodegenerative diseases [5]. However, in chronic obstructive pulmonary disease (COPD), mitophagy has been shown to involve pulmonary epithelial cell death [6], which indicates that mitophagy may serve as an effector of cell death. These results suggest the paradoxical role of mitophagy in diseases may depend on the regulation of cell death by mitophagy. Recently, various forms of RCD have been identified, such as apoptosis, pyroptosis, necroptosis, ferroptosis, parthanatos, entotic cell death, and NETotic cell death [7]. Increasing evidence has suggested the regulatory role of mitophagy in different forms of RCD [6, 8–11]. Therefore, it is necessary to provide a timely summary of research in this field, which will help researchers better understand the relationship between mitophagy and RCD and the role of mitophagy in the pathogenesis of diseases. In this review, we provided an overview of the regulatory role of mitophagy in RCD including autophagic cell death (ACD), apoptosis, pyroptosis, necroptosis and ferroptosis, and examined the implications of mitophagy-related RCD in diseases.

## The regulatory roles of mitophagy in RCD

### Mitophagy in autophagic cell death

ACD is a form of RCD that critically relies on the autophagy machinery. ULK1 complex, PI3K-III complex and two ubiquitin-like conjugation systems play a critical role in the regulation of autophagy [12]. As a form of selective autophagy, mitophagy can induce ACD when there is excessive clearance of mitochondria.

**Fig. 2:**
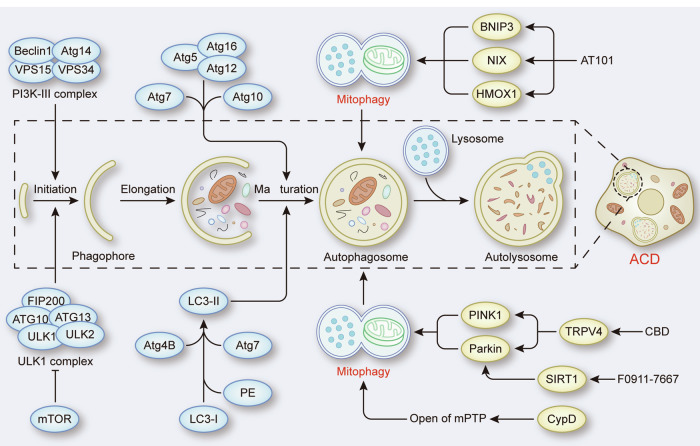
The role of mitophagy in ACD. ACD is characterized by the formation of autophagosomes and autolysosomes, which is a dynamic biological process that includes initiation, elongation, maturation of the phagophore and the fusion of autophagosomes and lysosomes. Mitophagy can be activated by the PINK1-Parkin pathway or mitophagy receptors such as BNIP3 and NIX to induce ACD. In addition, CypD-mediated opening of the mPTP plays a critical role in mitophagy-induced ACD.

During the treatment of glioma, certain drugs or compounds have been shown to target mitochondria, resulting in excessive mitophagy and ACD [13–15]. PINK1-Parkin-mediated mitophagy is the primary form of autophagy in cannabidiol (CBD)-induced ACD, in which the activation of TRPV4 plays a critical role in the initiation of mitophagy [13]. Likewise, activation of sirtuin-1 (SIRT1) by the compound F0911-7667 promotes PINK1-Parkin-mediated mitophagy to induce ACD, and the difference is that autophagy initiated by the former depends on the AKT-mTOR signaling pathway, whereas autophagy initiated by the latter depends on the AMPK-mTOR signaling pathway [13, 14]. Notably, it has been shown that the expression levels of Parkin are very low or even absent in many cancers, including gliomas [16, 17]. Therefore, it is necessary to identify alternative mechanisms by which mitophagy can induce ACD in these cases. In this context, BNIP3 and NIX can act as alternative mitophagy receptors to induce ACD without the presence of Parkin to promote mitophagy. The polyphenolic compound (-)-gossypol, which is also known as AT101, was shown to promote the expression of BNIP3 and NIX and upregulated heme oxygenase-1 (HMOX1), thus triggering lethal mitophagy and contributing to AT101-induced ACD [15]. In addition, experiments on aging models highlight the role of the mitochondrial permeability transition pore (mPTP) in mitophagy-induced ACD. In *Podospora anserina*, the abundance of CypD was increased during aging, and the reduction in lifespan in the strain overexpressing CypD relied on ACD induced by mitophagy, which was mediated by opening of the mPTP [18]. Similarly, ACD induced by impairment of F_1_F_0_-ATP synthase has been shown to be caused by an increase in mitophagy, which is associated with CypD-mediated opening of the mPTP in *P. anserina* [19] (Fig. 2).

### Mitophagy in apoptosis

#### Mitophagy inhibits apoptosis

Apoptosis is the earliest identified RCD and intrinsic apoptosis is the primary form of apoptosis regulated by mitophagy. BCL2 family members are important molecules that link mitophagy and intrinsic apoptosis. In diabetic retinopathy, mitophagy activation mediated by PINK1-Parkin reverses high-glucose (50 mM)-induced retinal pigment epithelium apoptosis, which is achieved by upregulating BCL2 and downregulating BAX [20]. Similar results have been confirmed in different models. In hepatocytes treated with copper exposure and neurons exposed to IRI, the activation of PINK1-Parkin-mediated mitophagy was shown to negatively regulate apoptotic cell death by increasing the ratio of BCL2 to BAX or BAK [8, 21]. Overall, the mitophagy-mediated upregulation of antiapoptotic proteins and downregulation of proapoptotic proteins leads to an increase in the antiapoptotic signal relative to the proapoptotic signal, which inhibits the release of cytochrome c, and prevents apoptosis by inactivating caspase-9/3.

Mechanistically, selective escape of outer mitochondrial membrane (OMM) proteins from mitochondria during mitophagy may lead to the upregulation of antiapoptotic proteins and promote cell survival. Depending on the ubiquitylation activity of Parkin, BCL2 and the antiapoptotic protein FKBP38 translocate from the mitochondria to the ER during Parkin-dependent mitophagy, thereby avoiding autophagosomal degradation [22]. Further research identified FKBP38 as a novel mitophagy receptor and showed that FKBP38 could avoid degradation during Parkin-independent mitophagy by escaping from mitochondria [23]. Conversely, the degradation of proapoptotic proteins by mitophagy is another mechanism by which mitophagy inhibits apoptosis. In DM platelets, hyperglycemia leads to the phosphorylation of p53, which translocates to mitochondria and is physically removed by mitophagy [24]. Subsequently, a reduction in the phosphorylation of p53 can protect cells from intrinsic apoptosis [24].

#### Mitophagy promotes apoptosis

**Fig. 3:**
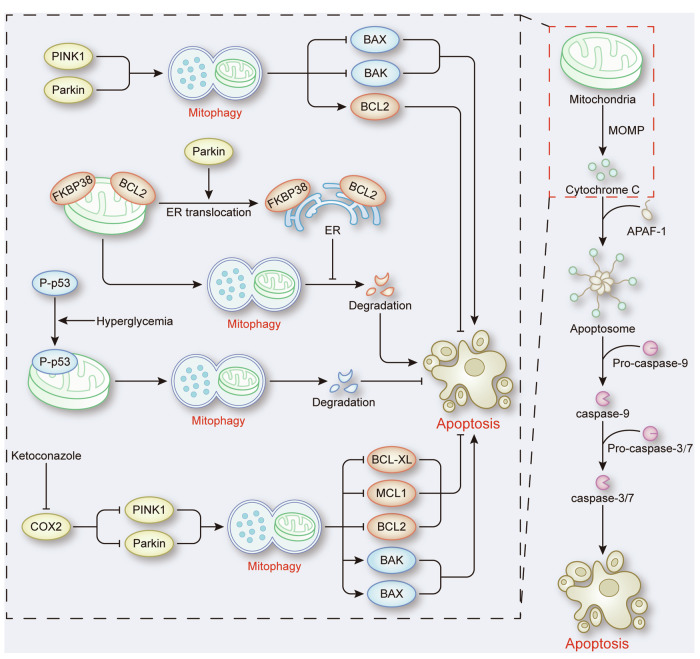
The role of mitophagy in intrinsic apoptosis. Mitochondrial outer membrane permeabilization (MOMP) facilitates the release of cytochrome C from mitochondria, thereby triggering caspase-dependent intrinsic apoptosis. During this process, mitophagy is involved in regulating intrinsic apoptosis by modulating the expression of BCL2 family members, as well as FKBP38 and the phosphorylation of p53.

Generally, mitophagy can remove damaged mitochondria to prevent apoptosis. However, there have been studies that show the positive regulatory effect of mitophagy on apoptosis. In hepatocellular carcinoma (HCC) cells, ketoconazole downregulates the expression of COX2, thereby triggering PINK1-Parkin-mediated mitophagy to enhance apoptosis, downregulate BCL2, BCL-XL and MCL1 and upregulate BAX and BAK [25] (Fig. 3). Additionally, in human gastric adenocarcinoma, 8-paradol exacerbates PINK1-Parkin-mediated mitophagy to decrease the expression of BCL2, whereas it increases the expression levels of BAX, cytochrome c, and caspase-9/3, ultimately inducing intrinsic apoptosis [26]. These studies attributed the induction of apoptosis by mitophagy to the excessive activation of mitophagy, which may result in excessive degradation of functional mitochondria, disrupting mitochondrial homeostasis and promoting apoptotic cell death. Although this viewpoint is plausible, the precise molecular mechanisms still require further exploration to better elucidate the regulation of apoptosis by mitophagy.

#### The potential reasons for dual regulatory role of mitophagy on apoptosis

The dual roles of mitophagy in regulating apoptosis may depend on the distinct roles played by mitophagy-associated molecules in apoptosis. Taking PINK1-Parkin-mediated mitophagy as an example, protein posttranslational modifications mediated by PINK1 and Parkin contribute to the complexity of apoptosis regulation by mitophagy. In response to mitochondrial damage, PINK1 can phosphorylate BCL-XL and BAD. The phosphorylation of BCL-XL prevents its proapoptotic cleavage, and the phosphorylation of BAD increases the threshold for mitochondrial apoptosis, both of which inhibit apoptosis [27, 28]. Similarly, Parkin can inhibit apoptosis through the ubiquitination of the substrates BAK and BAX. The ubiquitination of BAK at lys113 impairs its activation, oligomerization and apoptotic activity [29]. The ubiquitination of BAX limits its translocation from the cytosol to mitochondria to inhibit its proapoptotic function [30]. Notably, Parkin activation has been reported to promote apoptosis. The proteasomal degradation of MCL1, which is targeted by Parkin, may be one of the factors that induces apoptosis following mitochondrial damage, and this effect can be enhanced by BCL2-associated athanogene 5 (BAG5) and PINK1 [31–33]. It is clear that the balance between the ubiquitination and phosphorylation levels of BCL2 family proteins plays a crucial role in the regulation of apoptosis. Regarding mitophagy receptors, BCL2-like protein 13 (BCL2L13) shows dual regulatory effects similar to those of Parkin. In SF767 cells, BCL2L13 binds to ceramide synthase 2/6 (CERS2/6), which inhibits the oligomerization of BAX to prevent apoptosis [34]. In contrast, BCL2L13 exhibits proapoptotic activity by interacting with adenine nucleotide translocator (ANT), which increases the opening of mPTP to promote cytochrome c release in PC3 cells [35]. In addition, a recent study identified BCL-B, which is a newly discovered member of the BCL2 family that can induce mitophagy and seems to exert antiapoptotic or proapoptotic activity depending on the cellular context [36, 37]. Although these processes have been confirmed not to interfere with the mitophagy pathway, they can serve as upstream pathways of apoptotic signaling, thereby influencing the regulatory effect of mitophagy on cellular apoptosis. Further research is required to investigate how these processes mutually regulate each other and coordinate with mitophagy to regulate apoptosis.

Interestingly, a recent study revealed a potential connection between mitophagy and extrinsic apoptosis. Specifically, the OMM protein TUFM appears to inhibit caspase-8-mediated apoptosis by inducing mitophagy, playing a critical role in host antiviral responses and carcinogenesis [38]. Additionally, Parkin-mediated mitophagy was shown to promote BAX/BAK-independent release of cytochrome c from mitochondria to trigger caspase-9-dependent apoptosis, which is a noncanonical apoptotic process mediated by proteasome-dependent OMM degradation [39]. Therefore, it is necessary to conduct further research to explore the mechanisms by which mitophagy regulates different apoptotic pathways, which will help advance our understanding of the relationship between mitophagy and apoptosis.

### Mitophagy in pyroptosis

#### Mitophagy in NLRP3 inflammasome-mediated pyroptosis

**Fig. 4:**
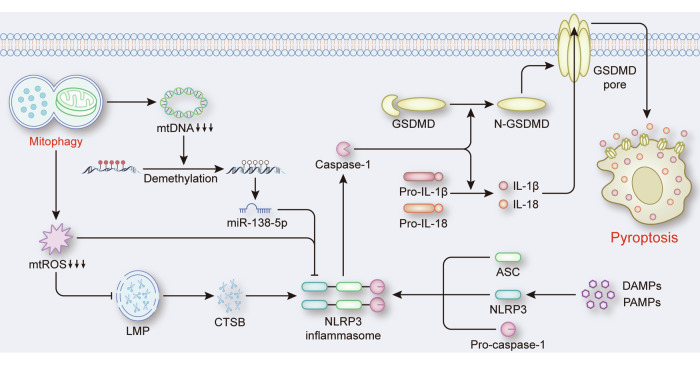
The role of mitophagy in NLRP3 inflammasome-mediated pyroptosis. DAMPs or PAMPs trigger the assembly of the NLRP3 inflammasome, thereby leading to the activation of caspase-1 and the subsequent cleavage of GSDMD, pro-IL-1β, and pro-IL-18. N-GSDMD forms pores in the plasma membrane to promote pyroptosis, which is accompanied by the release of pro-IL-1β, and pro-IL-18. During this process, mitophagy reduces the levels of mtDNA and mtROS, which inhibits the assembly of the NLRP3 inflammasome and subsequently prevents pyroptosis.

Pyroptosis is a form of inflammation-related RCD relies on inflammasome assembly [40]. Growing evidence shows that mitophagy is a negative regulatory factor of pyroptosis, especially NLRP3 inflammasome-mediated pyroptosis. Mechanistically, because mitochondrial ROS (mtROS) are a critical signal for NLRP3 inflammasome activation, moderate mitophagy levels inhibit pyroptosis by reducing the production of mtROS through the elimination of damaged mitochondria. In nonalcoholic steatohepatitis, liraglutide can induce PINK1-Parkin-mediated mitophagy, which restricts the generation of ROS to suppress NLRP3-mediated hepatocyte pyroptosis [41]. In spinal cord injury, betulinic acid significantly increases the expression levels of Parkin, BNIP3, and NIX to enhance mitophagy, which attenuates the accumulation of ROS and inhibits NLRP3-mediated pyroptosis [42]. Additionally, ROS can indirectly trigger pyroptosis by stimulating lysosomal membrane permeabilization (LMP), as the release of cathepsin B (CTSB) into the cytoplasm via LMP has been identified as a novel mechanism for activating NLRP3 inflammasomes [43, 44]. Mitophagy can inhibit pyroptosis through the ROS-CTSB pathway, which has been confirmed. In AML12 cells, apigenin activates mitophagy to clear ROS, which reduces LMP and CTSB release, thereby alleviating NLRP3 inflammasome activation [10]. Moreover, apart from mtROS, mitochondrial DNA (mtDNA) has become a key molecule for mitophagy-mediated regulation of pyroptosis. It has been reported that mitophagy-mediated decreases in the levels of cytoplasmic mtDNA lead to demethylation of the miR-138-5p promoter in sepsis-associated acute lung injury (ALI) [45]. This increases the expression of miR-138-5p, which targets NLRP3 to inhibit pyroptosis [45] (Fig. 4).

Interestingly, AIM2 and NLRP3 inflammasome-mediated pyroptosis leads to caspase-1-dependent inhibition of mitophagy [46], which reveals an interaction between pyroptosis and mitophagy. Further research confirmed the reciprocal inhibitory effect between pyroptosis and mitophagy. In alveolar macrophages, PINK1-mediated mitophagy has been proven to clear damaged mitochondria and negatively regulate pyroptosis, while inhibiting pyroptosis with NLRP3, caspase-1, and gasdermin D (GSDMD) inhibitors enhances PINK1-mediated mitophagy and reduces mitochondrial damage [47]. Through this kind of interaction, NLRP3 inflammasome-mediated pyroptosis is further enhanced, leading to the amplification of inflammation. In contrast, promoting mitophagy effectively suppresses the occurrence of pyroptosis. Notably, a recent study examined type I primary alveolar epithelial cells and showed that exposure to ozone increases cellular ROS levels, which activate PINK1-Parkin-mediated mitophagy and further promote NLRP3 inflammasome-mediated pyroptosis [48]. This finding challenges the notion of a mutual inhibitory effect between mitophagy and pyroptosis, suggesting the existence of other pathways linking mitophagy and pyroptosis that can disrupt the feedback mechanisms between these processes.

#### Mitophagy in non-NLRP3 inflammasome-mediated pyroptosis

Previous studies suggested that apoptosis-related caspases cannot stimulate gasdermin to induce pyroptosis; however, current research has shown that caspase-3/8 can cleave different members of the gasdermin family, such as GSDMC, GSDMD, and GSDME [49–51]. Given the crosstalk between apoptosis and pyroptosis established by caspase-3/8, apoptosis may serve as an intermediate link between mitophagy and pyroptosis. Indeed, there have been studies revealing novel mitophagy-pyroptosis regulatory pathways, such as the caspase-3/GSDME axis and caspase-9/GSDME axis, both of which are dependent on apoptosis [52, 53]. These discoveries suggest that other pyroptosis pathways may also be regulated by mitophagy through different mechanisms, which have been overlooked thus far. Therefore, further research is needed to explore this possibility.

### Mitophagy in necroptosis

#### Mitophagy inhibits necroptosis

Similar to their role in pyroptosis, mtROS are involved in signal transduction in necroptosis. It has been reported that mtROS can promote autophosphorylation of RIPK1 to recruit RIPK3, resulting in the formation of necrosomes to promote necroptosis [54]. Therefore, clearing mtROS through mitophagy may be a mechanism by which mitophagy inhibits necroptosis. In alcoholic liver disease, tetramethylpyrazine (TMP)-induced activation of PINK1-Parkin-mediated mitophagy has been reported to reduce the generation of mtROS, which inhibits the activation of necrosomes to suppress MLKL-dependent necroptosis [55]. In contrast, inhibition of PINK1-Parkin-mediated mitophagy by 1,3-dichloro-2-propanol (1,3-DCP) can induce renal tubular cell necroptosis through the ROS/RIPK3/MLKL axis [56]. In addition, studies have shown that mPTP opening signaling pathways can be inhibited by activating mitophagy [57–59], which may represent another mtROS-independent mechanism by which mitophagy inhibits necroptosis. A recent study demonstrated that in myocardial infarction, the upregulation of RIPK3 inhibits Parkin-mediated mitophagy, which increases the opening of the mPTP to induce necroptosis [58]. Mechanistically, this may be related to Parkin-mediated ubiquitination of CypD, a key component of the mPTP complex, which inhibits the opening of the mPTP [60]. Moreover, under necroptotic conditions, Parkin can interact with the N-terminus of RIPK3 via its IBR-R2 domain to disrupt the phosphorylation of RIPK3 while promoting its ubiquitination, thereby blocking the formation of necrosomes to inhibit necroptosis [61]; however, there is insufficient evidence to support the role of Parkin-mediated mitophagy in this necroptotic process and further research is needed to explore the potential relationship between Parkin-mediated mitophagy and Parkin-mediated ubiquitination of RIPK3.

#### Mitophagy induces necroptosis

**Fig. 5:**
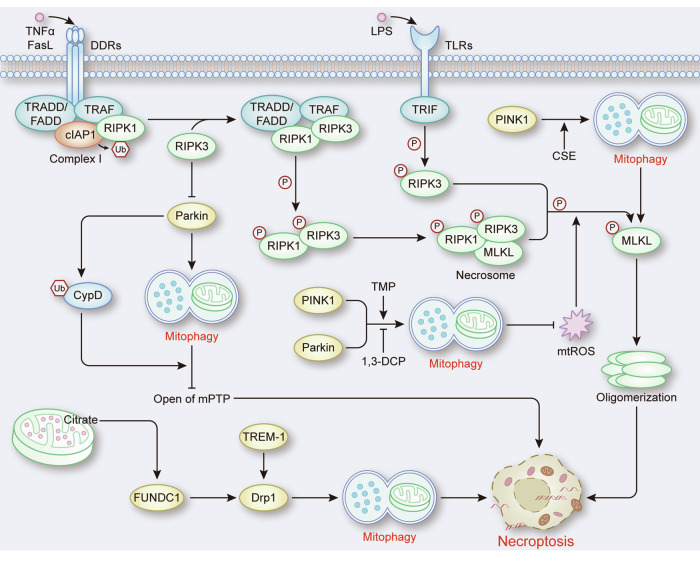
The role of mitophagy in necroptosis. The activation of death domain receptors (DDRs) and toll-like receptors (TLRs) promotes the assembly of the necrosome, which induces the oligomerization of MLKL to trigger necroptosis. During this process, mitophagy can inhibit necroptosis by preventing the opening of the mPTP and reducing the levels of mtROS to suppress MLKL-dependent necroptosis. However, PINK1-mediated MLKL phosphorylation during mitophagy and excessive mitophagy induced by Drp1 have been shown to promote necroptosis.

Numerous studies have suggested that mitophagy has a positive regulatory effect on necroptosis. In ALI, the accumulated mitochondrial citrate interacts with FUNDC1 and promotes the interaction of FUNDC1 with dynamin-related protein 1 (Drp1), which leads to excessive mitophagy to induce necroptosis [62]. Similarly, TREM-1 has been reported to activate Drp1-mediated mitochondrial fission, which subsequently triggers excessive mitophagy to induce necroptosis during ALI [63]. These results revealed the role of unchecked mitophagy in necroptosis. It is worth noting that while excessive mitophagy is widely recognized as a critical factor leading to cell death via mitophagy-related apoptosis and pyroptosis, a previous study indicated that extensive mitochondrial depletion via mitophagy did not affect necroptotic cell death [64], which raises the possibility that other factors promote necroptosis. During the pathogenesis of COPD, increased phosphorylation of MLKL, which partially results from PINK1-mediated mitophagy induced by cigarette smoke exposure (CSE), promotes pulmonary epithelial cell necroptosis [6] (Fig. 5). In response to this finding, a hypothesis has been proposed that activated mitophagy may aggravate mitochondrial damage and depolarization in the context of CSE-induced cell stress [6], which needs further research verification. In addition, in microglia, knockdown of Parkin seems to indirectly increase the ubiquitination level of RIPK1 to protect against necroptosis [65], which may increase the complexity of the interaction between mitophagy and necroptosis.

In brief, mitophagy is a crucial process that regulates necroptosis, and the regulatory effect of mitophagy on necroptosis depends on multiple factors, including the degree of mitophagy and the target of Parkin. It is clear that there is a complex interaction between mitophagy and necroptosis, and further research is needed to explore the underlying mechanisms of this interaction.

### Mitophagy in ferroptosis

Ferroptosis is a form of iron-dependent RCD mediated by the excessive accumulation of lipid peroxides [66]. Numerous studies have shown the positive regulatory effect of mitophagy on ferroptosis. The discovery of mitophagy-induced ferroptosis in melanoma cells is mediated by the inhibition of mitochondrial complex I by BAY 87-2243, which induces PINK1-dependent mitophagy to increase cellular ROS levels, thereby leading to the consumption of GSH and lipid peroxidation [67]. The mechanism by which cellular ROS accumulate is critical during mitophagy-mediated ferroptosis. Generally, mitophagy is thought to reduce the accumulation of mtROS, which is an important source of cellular ROS [68]. Interestingly, a study showed that mitophagy did reduce mtROS levels, but it was accompanied by an increase in cellular ROS and ultimately promoted ferroptosis [69]. Therefore, during mitophagy-induced ferroptosis, there are other pathways that increase cellular ROS levels.

Glutathione peroxidase 4 (GPX4) is an important antioxidant enzyme that protects cells from oxidative stress and lipid peroxidation by reducing the accumulation of ROS [70]. In fibrotic liver tissues, upregulated FUNDC1 interacts with GPX4 via its 96-133 amino acid domain to promote the translocation of GPX4 from the cytoplasm to mitochondria, where GPX4 is degraded along with damaged mitochondria by mitophagy, leading to the inhibition of ROS clearance and the induction of ferroptosis [11]. Notably, other important antioxidant molecules associated with ferroptosis, such as FSP1, GCH1, DHODH and VKH2, have garnered attention [66]. However, the relationship between these molecules and mitophagy in the context of ferroptosis has not yet been determined and requires further verification.

**Fig. 6:**
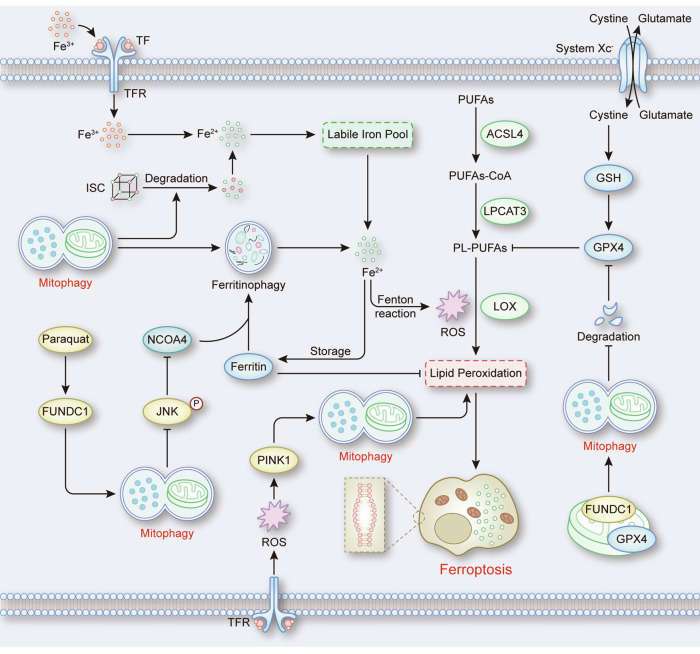
The role of mitophagy in ferroptosis. Iron overload promotes lipid peroxidation through the Fenton reaction, while the system Xc^−^-GPX4 pathway inhibits the formation of PL-PUFAs-OOH to suppress ferroptosis. During this process, mitophagy not only promotes ferritinophagy and ISC degradation to increase intracellular ferrous iron but also facilitates the degradation of GPX4, which elevates cellular ROS levels to promotes ferroptosis. In addition, activation of TFR elevates cellular ROS levels to active mitophagy, inducing lipid peroxidation and ferroptosis.

The increased generation of ROS induced by mitophagy mainly depends on the iron-mediated Fenton reaction. Mitophagy can participate in the increase in ferrous iron levels by releasing iron from mitochondrial iron‒sulfur clusters (ISCs) [71]. In addition, a study revealed that OSMI-1-mediated inhibition of *O*-GlcNAcylation induces ferritinophagy and mitophagy to jointly promote free ferrous iron release to induce ferroptosis, and inhibiting mitophagy by knocking down PINK1 partially prevented de-O-GlcNAcylation-induced ferroptosis [72]. Although it has been demonstrated that OSMI-1-induced ferritinophagy and mitophagy are relatively independent [72], research has suggested that there may be connections between mitophagy and ferritinophagy through other pathways. For example, the feedback mechanism of mitochondrial supplementation induced by mitophagy may activate ferritinophagy to provide adequate iron for the synthesis of new mitochondria [73]. Additionally, it has been shown that upregulation of the FUNDC1 mediated by paraquat exposure leads to the dephosphorylation of JNK, which increases the expression of nuclear receptor coactivator 4 (NCOA4) to promote ferritinophagy [74]. Overall, mitophagy not only induces the release of ferrous iron from mitochondria but also activates ferritinophagy to release ferrous iron from ferritin. These processes elevate cellular ferrous iron levels, which can react with H2O2 to generate ROS via the Fenton reaction, implicating in mitophagy-mediated ferroptosis. Notably, a study revealed that mitophagy could increase the level of mtROS [75], indicating an alternative pathway by which mitophagy increases cellular ROS levels. However, current research focuses more on changes in cellular total ROS levels during mitophagy-induced ferroptosis. The role of the increase in mtROS in mitophagy-induced ferroptosis warrants further investigation. In addition to serving as a bridge between mitophagy and ferroptosis, ROS can activate mitophagy to induce ferroptosis. For example, activation of TFR promotes the accumulation of ROS and overexpression of PINK1, which triggers mitophagy and leads to lipid peroxidation and ferroptosis [76] (Fig. 6). These results suggest ROS may have the potential to amplify mitophagy-ferroptosis signals through a positive feedback mechanism.

Interestingly, a recent study raised controversies about the role of mitophagy in ferroptosis by indicating that mitophagy could protect renal tubular epithelial cells from ferroptosis in cisplatin-induced acute kidney injury (AKI) [77]. Mechanistically, BNIP3- and PINK1-Parkin-mediated mitophagy reduce the excessive release of ROS and the expression of HMOX1, which abrogates the decrease in GPX4 and inhibits lipid peroxidation and ferroptosis [77]. Therefore, it is necessary to further explore mitophagy-mediated regulation of ROS to understand the role of mitophagy in ferroptosis.

## The implications of mitophagy-related RCD in diseases

Abnormal or excessive activation of cell death is a critical feature of various diseases such as tumors, neurodegenerative diseases, and cardiovascular diseases [78]. As previously mentioned, mitophagy plays a complex role in the regulation of different forms of RCD. By targeting mitophagy, it is possible to modulate the occurrence and extent of cell death, thereby influencing the progression of diseases and the degree of organ injury. Here, we summarize seven categories of diseases to elucidate the importance of mitophagy-related RCD in these diseases.

### Degenerative diseases

Mitophagy-related RCD is involved in the pathogenesis of degenerative diseases. Blood‒brain barrier (BBB) impairment and dysfunction are pathological changes in patients with Alzheimer’s disease (AD), and pericyte degeneration is the main cause [79]. Mechanistically, accumulated amyloid β (Aβ) proteins lead to BBB disruption by inducing pericyte mitophagy-mediated ferroptosis via the CD36/PINK1-Parkin pathway, which aggravates the pathological progression of AD [80]. Additionally, it has been shown that mitophagy disorder-mediated pyroptosis in microglia is one of the mechanisms by which copper exposure increases the risk of Parkinson’s disease (PD) [81]. During the pathogenesis of neurodegenerative diseases, activated microglia-mediated neuroinflammation plays a crucial role [82], and targeting mitophagy is an effective approach for inhibiting the activation of microglia. For example, in PD mouse models, quercetin (Qu) alleviates neurodegeneration by inhibiting NLRP3 inflammasome-mediated pyroptosis by promoting mitophagy in microglia [83].

**Fig. 7:**
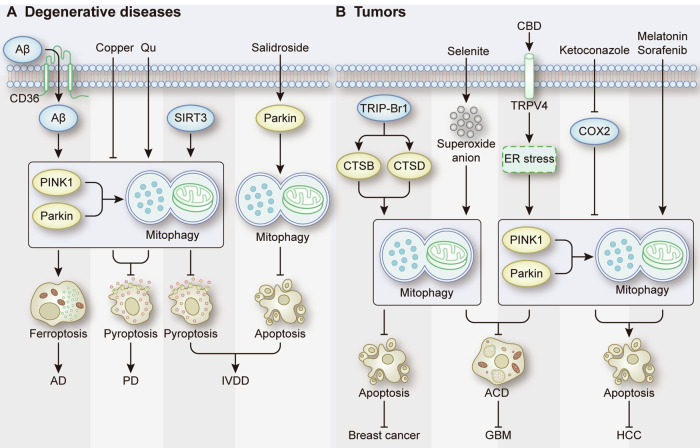
Mitophagy-related RCD in degenerative diseases and tumors. **A** The role of mitophagy-related RCD in AD, PD, and IVDD. **B** The role of mitophagy-related RCD in breast cancer, GBM and HCC.

In addition to neurodegenerative diseases, the role of RCD in degenerative osteoarthrosis cannot be ignored. Nucleus pulposus (NP) cells play a critical role in maintaining the biological and mechanical function of intervertebral discs [84]. In NP tissues from patients with intervertebral disc degeneration (IVDD), NLRP3 inflammasome activation promotes the release of inflammatory cytokines such as IL-1β, which is considered to be a key mediator of IVDD [85, 86]. During the progression of IVDD, NP cells exposed to inflammatory cytokines undergo apoptosis, leading to the loss of functional cells in the intervertebral disc [9, 87]. Accumulating evidence has shown that targeting mitophagy to inhibit RCD is a potential therapeutic strategy for IVDD. SIRT3 has been shown to trigger PINK1-Parkin-mediated mitophagy to inhibit IL-1β-induced NLRP3 inflammasome activation in NP cells [88]. A20 plays a self-protective role in NP cells through anti-inflammatory effects [89]. A study showed that overexpression of A20 could attenuate NP cell pyroptosis and apoptosis by promoting mitophagy [9]. Additionally, Animal experiments further confirm the feasibility of this therapeutic strategy. In a puncture-induced IVDD rat model, salidroside-induced upregulation of Parkin activated mitophagy to protect NP cells from apoptosis, which significantly ameliorated the progression of IVDD [90] (Fig. 7A). In summary, these results indicate that mitophagy is a potential target that can offer novel therapeutic strategies for the treatment of degenerative diseases.

### Tumors

In recent years, chemotherapy has become a widely used tumor treatment. Mechanistically, the toxicities of the majority of anticancer drugs are attributed to the induction of mitochondrial dysfunction, which results in cancer cell apoptosis [91, 92]. However, cancer cells may eliminate damaged mitochondria and escape apoptosis via mitophagy, which leads to the development of resistance. A study showed that following treatment with various anticancer drugs, upregulation of the TRIP-Br1 oncoprotein is one of the reasons for the development of drug resistance in breast cancer cells [93]. Increased TRIP-Br1 promotes mitophagy through upregulating CTSB and CTSD, which in turn inhibits cancer cell apoptosis and ultimately promotes cancer cell survival [93]. Therefore, targeting mitophagy to induce RCD in tumor cells represents a new therapeutic strategy that may help overcome chemotherapy resistance. On the one hand, treatment with anticancer drugs that induce nonapoptotic cell death by targeting mitophagy is a strategy to improve cancer cell sensitivity to chemotherapy. A recent study engineered a nanopyroptosis amplifier that contained lonidamine (LND) and black phosphorus nanosheets (BP) for the treatment of glioblastoma (GBM), and BP was used to block mitophagy flux and amplify LND-induced pyroptosis [94]. Additionally, sodium selenite is preferentially cytotoxic to human glioma cells relative to normal astrocytes by inducing Atg6- and Atg7-dependent ACD, and this cytotoxic effect relies on mitophagy facilitated by superoxide anions produced by selenite [95]. These findings provide a new approach to overcome resistance to temozolomide (TMZ), which is commonly used as the first-line chemotherapy agent for glioma. On the other hand, combining drugs targeting mitophagy with anticancer drugs is an effective approach for overcoming chemotherapy resistance. For example, CBD can effectively enhance the antitumor effect of TMZ on gliomas by promoting lethal mitophagy-induced ACD [13]. In addition, primary and acquired drug resistance are common occurrences in response to sorafenib, a first-line chemotherapeutic agent for HCC [96]. Studies have shown that combined treatment with ketoconazole or melatonin and sorafenib can significantly reduce sorafenib resistance in HCC cells [25, 97]. Mechanistically, ketoconazole and melatonin target PINK1-Parkin-mediated mitophagy to induce apoptosis, sensitizing HCC cells to sorafenib treatment [25, 97] (Fig. 7B). Moreover, it has been reported that cotreatment with liensinine to inhibit late-stage mitophagy increased the sensitivity of breast cancer cells treated with various chemotherapeutic agents, including doxorubicin, paclitaxel, vincristine, and cisplatin, to apoptosis [98].

### Drug and poison damage

Drug adverse reactions, particularly those associated with anticancer drugs, are difficult to avoid during clinical treatments. Mitophagy-related RCD has been reported to be a potential mechanism of drug toxicity. As a key tyrosine kinase inhibitor, sorafenib causes heart damage during the treatment of tumors [99]. A study indicated that sorafenib led to the degradation of mitofusin-2 (MFN2) in lysosomes by promoting PINK1-Parkin-mediated mitophagy in cardiomyocytes, and the decrease in MFN2 increased the expression of calmodulin-dependent protein kinase II delta (CaMKIIδ), thereby contributing to cardiac necroptosis [100]. Targeting mitophagy has been proven to be an effective strategy for reducing drug toxicity. Cisplatin is widely recommended for combination therapy in various tumors; however, it has been reported that approximately 20%-30% of patients develop AKI during cisplatin treatment [101]. Evidence has shown that promoting PINK1-Parkin or BNIP3-mediated mitophagy can alleviate cisplatin-induced AKI by inhibiting renal tubular epithelial cell ferroptosis [77]. Additionally, targeting mitophagy is effective for managing adverse reactions caused by steroid treatment. For example, osteonecrosis of the femoral head (ONFH) induced by steroids is an urgent problem in the field of orthopedics [102]. Bone marrow mesenchymal stem cells (BMSCs) transplantation is a treatment approach for steroid-induced ONFH; however, its efficacy is limited by apoptosis in transplanted BMSCs due to the oxidative stress microenvironment of the femoral-head necrotic area [103]. By downregulating the expression of p53 in BMSCs, mitophagy could be enhanced, which effectively inhibited BMSCs apoptosis and improved the outcomes of steroid-induced ONFH treated with BMSCs transplantation [104].

**Fig. 8:**
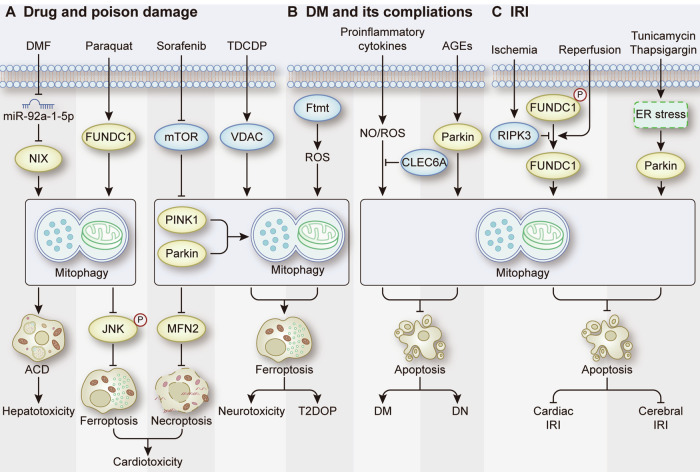
Mitophagy-related RCD in drug and poison damage, DM and its complications, and IRI. **A** The role of mitophagy-related RCD in DMF-induced hepatotoxicity, paraquat-induced cardiotoxicity, sorafenib-induced cardiotoxicity, TDCDP-induced neurotoxicity. **B** The role of mitophagy-related RCD in DM, T2DOP and DN. **C** The role of mitophagy-related RCD in cardiac IRI and cerebral IRI.

Similar to drug damage, many studies have suggested that RCD regulated by mitophagy is involved in the progression of toxic disease. Evidence has shown that FUNDC1-mediated mitophagy induces ferroptosis to contribute to the cardiotoxicity of paraquat [74]. In addition, the neurotoxicity of Tris (1,3-dichloro-2-propyl) phosphate (TDCDP) has been reported to be related to ferroptosis induced by PINK1-Parkin-mediated mitophagy [105]. Moreover, N,N-dimethylformamide (DMF) has been reported to downregulate miR-92a-1-5p, which targets NIX to promote lethal mitophagy and induce ACD, thereby contributing to the hepatotoxicity of DMF [106] (Fig. 8A). Overall, these findings provide promising therapeutic directions for treating drug damage and toxic diseases by targeting mitophagy to modulate RCD.

### DM and its complications

Insufficient pancreatic β cell function or mass is a common etiology of DM [107]. Evidence has shown the positive role of Parkin-dependent mitophagy in maintaining the metabolic function needed for the release of insulin by β cells [108, 109]. Additionally, a study showed that knockdown of CLEC16A, which encodes an E3 ubiquitin ligase that controls mitophagic flux in β cells, impaired mitophagy, leading to the aggravation of apoptosis in β cells treated with proinflammatory cytokines [110]. This result suggests that mitophagy can protect β cells from apoptosis, thereby promoting their survival and maintaining their mass in DM. Targeting mitophagy is an effective strategy for the treatment of DM. For example, the efficacy of silibinin for the treatment of type 2 DM (T2DM) has been verified by research that demonstrated that silibinin could ameliorate the inhibition of PINK1-Parkin-mediated mitophagy in INS-1 cells treated with palmitic acid and high glucose, thereby attenuating ferroptosis to rescue cell loss [111]. Moreover, in diabetic platelets, mitophagy can prevent platelet apoptosis and protect platelet function to reduce thrombus formation, which decreases the incidence and mortality of thrombotic cardiovascular events in DM [24]. In addition to DM, there have been studies suggesting the role of mitophagy-related RCD in complications of DM, such as type 2 diabetic osteoporosis (T2DOP) and diabetic nephropathy (DN). T2DOP is a common complication of T2DM and is associated with an imbalance in iron homeostasis caused by metabolic alterations in T2DM [112–114]. The imbalance in iron homeostasis may lead to the occurrence of ferroptosis, which has been shown to be related to the pathogenesis of T2DOP and involves mitophagy [115]. Mechanistically, FtMt deficiency induces PINK1-Parkin-mediated mitophagy, thereby promoting osteoblastic ferroptosis and leading to a decrease in osteogenic function in T2DOP [115]. Additionally, the formation and deposition of advanced glycation end products (AGEs) are accelerated during DM, which have been shown to promote the generation of ROS to induce mesangial cell apoptosis, thereby playing a crucial role in the pathogenesis of DN [116] (Fig. 8B). A study showed that in AGEs-treated mesangial cells, AGEs-induced apoptosis was aggravated by the inhibition of mitophagy, which indicates the protective role of mitophagy in DN [117].

### Ischemia‒reperfusion Injury

Ischemia**‒**reperfusion (IR) is a complex pathological process. The depletion of oxygen and ATP in the ischemic phase and the excessive inflammatory response during the reperfusion phase contribute to cell death and ultimately organ dysfunction [118]. Studies have shown that IR activates mitophagy. Following renal IR, mitophagy is activated in proximal tubular cells in mice [119]. In the cortex of mice subjected to transient middle cerebral artery occlusion (tMCAO), enhanced mitophagy was detected in neurons via electron microscopy [120]. Correspondingly, inhibiting mitophagy has been shown to aggravate apoptosis, leading to worse organ injury, which reveals the protective role of mitophagy in IRI [119, 120]. Notably, these two results ignored the impact of the ischemic phase on mitophagy. Specifically, a study focusing on heart IRI demonstrated that mitophagy was induced during the ischemia phase by FUNDC1 dephosphorylation to block apoptotic signals, while during the reperfusion phase, an increase in RIPK3 disrupted FUNDC1 activation to increase the likelihood of apoptosis [57], which could limit the protective effect of mitophagy. Therefore, activation of mitophagy can be a therapeutic strategy against IRI. Ischemic preconditioning (IPC) is one of the most effective interventions for protecting organs against subsequent IRI, and its protective e0ffects have been proven to be associated with mitophagy [121, 122]. A study showed that IPC promoted PINK1-Parkin-mediated mitophagy to prevent tubular cell apoptosis and subsequent renal IRI, while inhibiting mitophagy abolished the protective effects of IPC [122]. In addition, treatment with the endoplasmic reticulum (ER) stress activators tunicamycin and thapsigargin has been shown to induce Parkin-dependent mitophagy to alleviate neuronal apoptosis, thereby protecting against ischemic brain injury [123] **(**Fig. 8C**)**.

### Infectious diseases

Due to the emergence of antibiotic resistance, novel drugs need to be developed to treat bacterial infection. It has been reported that mitochondria play a crucial role in antibacterial immunity [124], which suggests that drugs targeting mitophagy may be promising candidates for the treatment of bacterial infection. RCD is involved in the antibacterial process of mitophagy. In *vitro*, purple sweet potato anthocyanins (PSPAs) activated NRF2 signaling pathway to enhance PINK1-Parkin-mediated mitophagy, attenuating *Klebsiella pneumoniae* (KP)-induced pyroptosis in alveolar macrophages, and in mice infected with KP, PSPA has been shown to alleviate lung injury and inflammatory responses [125]. These findings indicate that PSPA is a potential treatment option for KP infection.

**Fig. 9:**
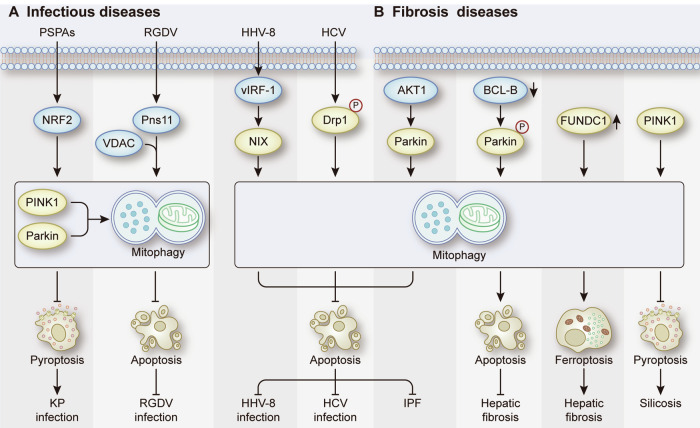
Mitophagy-related RCD in infectious diseases and fibrosis diseases. **A** The role of mitophagy-related RCD in KP infection, RGDV infection, HHV-8 infection, and HCV infection. **B** The role of mitophagy-related RCD in IPF, hepatic fibrosis, and silicosis.

In addition to antibacterial immunity, mitochondria participate in a broad range of innate immune responses to regulate antiviral signaling pathways [124]. However, during the coevolutionary process between pathogens and hosts, viruses have developed various strategies to counteract the immunological defenses of the host [126]. One of these mechanisms is the elimination of infection-induced mitochondrial changes via mitophagy, which inhibits cell apoptosis and innate immune responses. For example, human herpesvirus 8 (HHV-8) encodes vIRF-1, which is important for viral replication [127]. Following lytic reactivation, upregulated vIRF-1 in HHV-8-infected cells activated NIX-mediated mitophagy to inhibit apoptosis and promote productive viral replication, which plays a critical role in maintaining viral load within the host and for Kaposi’s sarcoma pathogenesis [128]. Additionally, the nonstructural protein Pns11 of rice gall dwarf virus (RGDV) has been shown to interact with voltage-dependent anion channel 1 (VDAC1) to activate proviral PINK1-Parkin-mediated mitophagy in leafhopper vectors [129]. This mechanism prevents extensive apoptotic responses in virus-infected regions and provides an optimal intracellular environment for persistent viral propagation. Moreover, persistent hepatitis C virus (HCV) infection has been reported to be associated with Parkin-dependent mitophagy in liver cells [130]. Mechanistically, HCV induced Drp1-mediated mitochondrial fission and subsequent mitophagy to attenuate apoptosis in infected cells, thereby facilitating virus secretion and evasion of innate immunity [130] **(**Fig. 9A**)**. In summary, the role of mitophagy-mediated regulation of apoptosis in viral infections provides a novel approach for the treatment of viral infectious diseases. Notably, the dysfunctional host response to infection can result in sepsis, a critical illness that poses a significant risk of life-threatening organ dysfunction [131]. Research has revealed the role of mitophagy-related RCD in organ injury induced by sepsis. In sepsis-associated AKI, BMSCs inhibited apoptosis and pyroptosis in renal tubular epithelial cells to ameliorate kidney injury by inducing Parkin-dependent mitophagy [132]. Similarly, activating mitophagy in alveolar macrophages holds promise as a therapeutic strategy for the treatment of ALI associated with sepsis by inhibiting pyroptosis [45].

### Fibrosis

Fibrosis is a pathological feature of most chronic inflammatory diseases and is characterized by the accumulation of excess extracellular matrix components and a decline in parenchymal cells [133]. In hepatic fibrosis, the deposited extracellular matrix proteins, particularly collagen I, primarily originate from activated hepatic stellate cells (HSCs) [134]. Therefore, clearance of HSCs is critical for preventing the progression of liver fibrosis. Knockdown of BCL-B enhances the phosphorylation of Parkin to induce mitophagy, thereby promoting apoptosis in HSCs [36]. Additionally, the upregulation of FUNDC1 was observed in the liver tissues of mice and patients with liver fibrosis, and it induced liver injury by promoting mitophagy-dependent ferroptosis in hepatocytes; conversely, knockdown of FUNDC1 effectively protected against CCl4-induced liver fibrosis in vivo [11]. These results provide new insights for the treatment of liver fibrosis. Apart from liver fibrosis, mitophagy-related RCD plays a crucial role in the pathogenesis of pulmonary fibrosis. In idiopathic pulmonary fibrosis (IPF), the activation of AKT1 in alveolar macrophages is required for the development of pulmonary fibrosis [135]. Mechanistically, AKT1 leads to the polarization of macrophages to a profibrotic phenotype; moreover, upregulated AKT1 induces Parkin-dependent mitophagy to contribute to profibrotic alveolar macrophage apoptosis resistance during fibrosis [135]. However, in the context of silicosis, the progression of pulmonary disease was associated with pyroptosis in alveolar macrophages mediated by mitochondrial damage, and PINK1-mediated mitophagy was shown to alleviate crystalline silica-induced pyroptosis [47] (Fig. 9B). The contradictory roles of mitophagy in alveolar macrophages of fibrotic lungs may depend on the involvement of distinct signaling pathways and disease contexts. Further research will help us better understand the pathogenesis of pulmonary fibrosis and provide novel targets and therapeutic strategies for its treatment.

## Conclusions and perspectives

In this review, we comprehensively discussed how mitophagy regulates different forms of RCD, and elucidated the role of mitophagy-related RCD in different diseases. Numerous studies have demonstrated the therapeutic potential of strategies targeting mitophagy to regulate RCD, and related drugs have shown promise in the treatment of various diseases (Table 1). However, there are four key unanswered questions that need to be addressed by further research.

**Table 1:** Potential drugs that regulate RCD by targeting mitophagy in diseases.

Drugs	Structural formula	Molecular weight	Molecular mechanism	Molecular function	IC50	In vitro model	In Vivo model	Diseases	Clinical trail	Ref.
Compounds targeting ACD										
CBD		258.36	Inducing PINK1-Parkin-mediated mitophagy	Inducing ACD	20‒30 μM	A172, U251,U87 MG, U118 MG and LN18 cells	Nude mice	Glioma	Phase 2	[13]
F0911-7667		535.03	Inducing PINK1-Parkin-mediated mitophagy	Inducing ACD	Not described	U87MG and T98G cells	BALB/c mice	Glioblastoma	Not Found	[14]
AT101		518.55	Inducing BNIP3 and NIX-mediated mitophagy	Inducing ACD	Not described	MZ-54, U87MG and U343 cells	None	Glioma	Phase 2	[15]
Compounds targeting apoptosis										
Ketoconazole		531.43	Inducing PINK1-Parkin-mediated mitophagy	Inducing apoptosis	Not described	HepG2, Hep3B and LO2 cells	PDX model	HCC	Not Found	[25]
Melatonin		232.28	Inducing PINK1-Parkin-mediated mitophagy	Inducing apoptosis	Not described	Hep3B cells	None	HCC	Not Found	[138]
8-paradol		306.44	Inducing PINK1-Parkin-mediated mitophagy	Inducing apoptosis	Not described	AGS cells	None	Human gastric adenocarcinoma	Not Found	[26]
Liensinine		610.74	Inhibiting late-stage mitophagy	Inducing apoptosis	Not described	MDA-MB-231 and MCF-7 cells	None	Breast cancer	Not Found	[98]
Salidroside		300.30	Inducing Parkin-dependent mitophagy	Inhibiting apoptosis	Not described	NP cells	SD rats	IVDD	Not Found	[90]
Tunicamycin		844.94	Inducing Parkin-dependent mitophagy	Inhibiting apoptosis	Not described	Primary neuronal cell cultures and Neuro2a cells	C57BL/6 mice	Ischemic brain injury	Not Found	[123]
Thapsigargin		650.75	Inducing Parkin-dependent mitophagy	Inhibiting apoptosis	Not described	Primary neuronal cell cultures and Neuro2a cells	C57BL/6 mice	Ischemic brain injury	Not Found	[123]
Compounds targeting pyroptosis										
Liraglutide		3751.20	Inducing PINK1-Parkin-mediated mitophagy	Inhibiting pyroptosis	Not described	HepG2 cells	None	Nonalcoholic steatohepatitis	Phase 4	[41]
Betulinic acid		456.70	Inducing mitophagy mediated by Parkin, NIX and BNIP3	Inhibiting pyroptosis	Not described	None	C57BL/6 mice	Spinal cord injury	Not Found	[42]
Quercetin		302.24	Inducing PINK1-Parkin-mediated mitophagy	Inhibiting pyroptosis	Not described	BV2 cells	C57BL/6 mice	PD	Not Found	[83]
PSPAs		597.15	Inducing PINK1-Parkin-mediated mitophagy	Inhibiting pyroptosis	Not described	Alveolar macrophages	C57BL/6 mice	KP infection	Not Found	[125]
Compounds targeting necroptosis or ferroptosis										
TMP		136.19	Inducing PINK1-Parkin-mediated mitophagy	Inhibiting necroptosis	Not described	LO2 cells	ICR mice	Alcoholic liver disease	Not Found	[55]
BAY 87-2243		525.53	Inducing PINK1-dependent mitophagy	Inducing necroptosis and ferroptosis	G361 cells: 4.8 ± 0.45 nm; SK-MEL-28 cells: 2.4 ± 0.86 nm	G361 and SK-MEL-28 cells	None	Melanoma	Not Found	[67]
Silibinin		482.44	Inducing PINK1-Parkin-mediated mitophagy	Inhibiting ferroptosis	Not described	INS-1 cells	None	T2DM	Not Found	[111]

First, numerous studies indicated that excessive mitophagy may promote RCD [25, 26, 62, 63]; however, there is no scientific basis for how to assess the degree of mitophagy, therefore, further research is needed to investigate whether objective metrics, such as morphological or biochemical indicators, are available to identify the degree of mitophagy. Second, in apoptosis and necroptosis, mitophagy-related molecules could modify BCL2 family proteins and RIPK3, respectively [27–29, 61]; further research is needed to confirm whether they also modify key proteins involved in other cell death pathways such as pyroptosis and ferroptosis, and the role of these modifications in the regulation of RCD by mitophagy. Third, the emergence of PANoptosis, a novel inflammatory RCD linking pyroptosis, apoptosis and necroptosis [136], suggests an intrinsic crosstalk between different forms of RCD. Although mitophagy has been shown to simultaneously regulate multiple forms of RCD, including apoptosis, pyroptosis and ferroptosis [9, 137], the intrinsic link between them has not been fully elucidated and is a potential target for further research. Finally, while there is limited research on the relationship between mitophagy and other forms of RCD, such as parthanatos, alkaliptosis, and NETosis, evidence suggests that mitochondria play a critical role in regulating these types of RCD [78]. This finding underscores the potential role of mitophagy in the regulation of these types of RCD, and further research on this topic would be valuable. Answering these questions will not only further our understanding of mitophagy but also provide a more precise interpretation of the regulatory mechanism of mitophagy in RCD to offer more effective guidance for treating related diseases.

## Data Availability

All data analyzed during this study are included in this published article. All data are available from the corresponding author on reasonable request.
